# A multicenter study of incidence, risk factors and outcomes of babies with birth asphyxia in Nepal

**DOI:** 10.1186/s12887-021-02858-y

**Published:** 2021-09-10

**Authors:** Avinash K. Sunny, Prajwal Paudel, Jagannath Tiwari, Bishow Bandhu Bagale, Antti Kukka, Zhou Hong, Uwe Ewald, Sara Berkelhamer, Ashish KC

**Affiliations:** 1 Golden Community, Lalitpur, Nepal; 2 Ministry of Health and Population, Kathmandu, Nepal; 3 Department of Women’s and Children’s Health, Uppsala University, Uppsala, Sweden; 4 Department of Paediatrics, Länssjukhuset Gävle-Sandviken, Gävle, Sweden; 5 Department of Maternal and Child Health, Peking University of Health Sciences, Peking, China; 6 Department of Pediatrics, University of Washington, Seattle, WA USA

**Keywords:** Newborn, Birth asphyxia, Risk factor, Neonatal mortality, Nepal

## Abstract

**Background:**

Perinatal events which result in compromised oxygen delivery to the fetus can lead to Birth Asphyxia (BA). While the incidence, risk factors and outcomes of BA have been characterized, less is known in low resource settings.

**Aim:**

To determine the incidence of Birth Asphyxia (BA) in Nepal and to evaluate associated risk factors and outcomes of this condition.

**Methods:**

A nested observational study was conducted in 12 hospitals of Nepal for a period of 14 months. Babies diagnosed as BA at ≥37 weeks of gestation were identified and demographics were reviewed. Data were analyzed using binary logistic regression followed by multiple logistic regression analysis.

**Results:**

The incidence of BA in this study was 6 per 1000 term livebirths and was higher among women 35 years and above. Predictors for BA were instrumented vaginal delivery (aOR:4.4, 95% CI, 3.1–6.1), fetal distress in labour (aOR:1.9, 95% CI, 1.0–3.6), malposition (aOR:1.8, 95% CI, 1.0–3.0), birth weight less than 2500 g (aOR:2.0, 95% CI, 1.3–2.9), gestational age ≥ 42 weeks (aOR:2.0, 95% CI, 1.3–3.3) and male gender (aOR:1.6, 95% CI, 1.2–2.0). The risk of pre-discharge mortality was 43 times higher in babies with BA (aOR:42.6, 95% CI, 32.2–56.3).

**Conclusion:**

The incidence of Birth asphyxia in Nepal higher than in more resourced setting. A range of obstetric and neonatal risk factors are associated with BA with an associated high risk of pre-discharge mortality. Interventions to improve management and decrease rates of BA could have marked impact on outcomes in low resource settings.

## Introduction

Globally, every year among the 140 million neonates, 10–15 million do not cry or breathe at birth [1–3]. These babies require resuscitation to transit from intra-uterine to the extra-uterine environment [4]. Neonates who do not receive timely and adequate resuscitation either die or develop brain injuries and long term disability [5]. Every year, it is estimated that one million neonates die due to intrapartum-related complication, also known as “birth asphyxia” [6]. Two million neonates have hypoxic ischemic encephalopathy and 1.2 million have developmental delays [5]. Most of the deaths take place in low and middle income countries.

Birth asphyxia can result from limited oxygen flow to the brain and impaired cerebral blood flow at the time of birth. It can later manifest as cerebral palsy and/or impaired cognitive, behavioural and motor development [7]. The hypoxic insult from BA can result in irreversible CNS injury including necrosis and persistent inflammation, potentially leading to long-term sequalae or non-survival [8]. Preventing the original insult or injury has potential to both reduce associated mortality and risks of long-term impairment [9].

In low and middle income settings, access to antenatal and intrapartum care may be challenged compromising obstetric management and risking intrapartum insults [10]. Several studies have evaluated intrapartum factors as well as obstetric and fetal conditions associated with BA [11–13]. Early and effective intrapartum management of obstetric and fetal complication can prevent BA [14]. For babies who develop BA, improved intrapartum and postnatal care can reduce mortality and decreased risk of associated developmental delay [15–17].

Use of a neonatal resuscitation protocol has been scaled up in health facilities in Nepal to address the management of non-breathing babies [18, 19]. However, challenges remain in improving the quality of neonatal resuscitation in these low resource settings [20]. The poor quality of neonatal resuscitation coupled with suboptimal obstetric and postnatal care pose a risk for BA. We conducted a study to evaluate the impact of use of neonatal resuscitation program on health workers’ performance and birth outcomes in 12 hospitals of Nepal [21].

In this study, we aimed to assess the incidence of BA, associated obstetric and neonatal risk factors and mortality outcomes in the cohort of affected infants.

## Methods

### Study design and setting

This was a nested study within a large-scale observational study [21] to evaluate the health worker’s performance on neonatal resuscitation and its birth outcome in 12 hospitals in Nepal. The study was conducted for a period of 14 months from July 1, 2017 to August 29, 2018. The included hospitals are government funded referral centers providing obstetric, neonatal and pediatric services and spread throughout the country. The records from these hospitals suggest that number of deliveries per hospital ranged from 1065 to 11,318 annually (Table 1). In these hospitals, all the babies are given Apgar Score at 1 and 5 min after birth. Babies with low Apgar score and those who received resuscitation are evaluated by pediatric team either in the delivery room or after being admitted in special newborn care unit (SNCU) or neonatal intensive care unit (NICU). Based on thorough evaluation of the experts in these units, babies are diagnosed as BA and provided with the supportive management.

**Table 1:** Annual delivery in the hospitals (year:2015)

Name of hospital	Total deliveries per year	Services available
Western Regional Hospital	9427	L&D + OT + SNCU
Mid-Western Regional Hospital	3139	L&D + OT + SNCU
Bardiya District Hospital	1065	L&D + SNCU
Bharatpur Hospital	11,318	L&D + OT + NICU
Seti Zonal Hospital	5767	L&D + OT + SNCU
Nuwakot District Hospital	1438	L&D + OT + SNCU
Koshi Zonal Hospital	8355	L&D + OT + SNCU
Rapti Sub-Regional Hospital	3280	L&D + SNCU
Nawalparasi District Hospital	1374	L&D + SNCU
Lumbini Zonal Hospital	9007	L&D + OT + NICU
Bheri Zonal Hospital	4276	L&D + OT + SNCU
Pyuthan District Hospital	1194	L&D + OT + SNCU

*L&D* Labour and Delivery room, *OT* Operation theatre, *SNCU* Special Newborn Care Unit, *NICU* Neonatal Intensive Care Unit

### Study participants

#### Inclusion criteria

- All babies born at gestational age ≥ 37 weeks within the study period at the selected hospitals were eligible for the study.
- Babies who had documented perinatal insult (Apgar Score < 7 at 5 min or non-breathing babies after birth) and admitted to the NICU/SNCU were included as birth asphyxia.

#### Exclusion criteria

- Babies who were outborn and had major congenital malformations

### Data collection and management

A data surveillance system was established in all the hospitals to collect data on maternal and newborn health. The socio-demographic and antenatal care information were assessed through semi-structured interviews at the time of discharge by data collectors. Clinical information on mothers and newborn were extracted in a data retrieval form. To ensure the privacy and safety of the data, the exported data was stored in external hard drive. For the data analysis purpose, the anonymization and removal of the location of the participants was maintained. For the purpose of this study, the following variables were extracted from the final database.

#### Outcome variables

- Birth Asphyxia- characterized as not breathing, gasping or Apgar score < 7 at 5 min of birth and admitted for special care.
- Pre-discharge mortality- death of the newborn before discharge from the hospital.

#### Demographic characteristics

- Maternal age- categorized into 15–19 years, 20–34 years and 35 years and above.
- Ethnicity- based on the Nepal’s caste based hierarchical system is categorized into Dalit, Janajati, Madhesi, Muslim, Chettri/Brahmin and others.

#### Obstetric characteristics

Induction of labour- the induction of labour using prostaglandin, artificial rupture of membrane and oxytocin.

Mode of delivery- spontaneous vaginal delivery, instrumented delivery and cesarean section.

Complication during labour-

- Multiple birth- more than 1 birth
- Suspected maternal infection- signs of clinical infection-fever and foul-smelling discharge and provided with prophylactic antibiotics.
- Fetal distress- abnormal (< 100 or > 160 beats per minute) or absent fetal heart rate for five minutes or more during labour and delivery
- Prolonged labour- labour lasting for 20 h or more for a primigravida and 14 h or more for multigravida.
- Malposition of fetus- abnormal positions of the vertex of fetal head relative to maternal pelvis.
- Prolapsed cord- the umbilical cord drops through the cervix into the vagina ahead of the baby.

#### Neonatal characteristics

##### Sex of the baby- male or female

Birthweight- categorized as < 2000 g, 2000-2499 g, 2500-4000 g and > 4000 g.

Meconium aspiration- baby born with meconium stained liquor who subsequently developed respiratory distress.

Gestational age- estimation of gestational age of babies using the last menstrual period, categorized into 37–41 weeks, 42 weeks or more.

### Statistical analysis

The incidence rate of BA was calculated by maternal age category and ethnicity. Binary logistic regression analysis was done for obstetric and neonatal factors to assess the level of association with BA. Variables which had missing values were excluded from the analysis. A *p*-value of < 0.05 was considered statistically significant. Variables with a *p-*value < 0.2 in the univariate analysis were considered for multiple logistic regression analysis.

### Ethical consideration

This study was approved by the Ethical Review Board of Nepal Health Research Council (NHRC). Written informed consent was taken from mothers before the exit interviews and confidentiality was maintained on the data obtained from extraction. For mother’s age under the 16 years’ informed consent was obtained from the legal guardians. All methods were carried out in accordance with relevant guidelines and regulations.

## Results

**Fig. 1:**
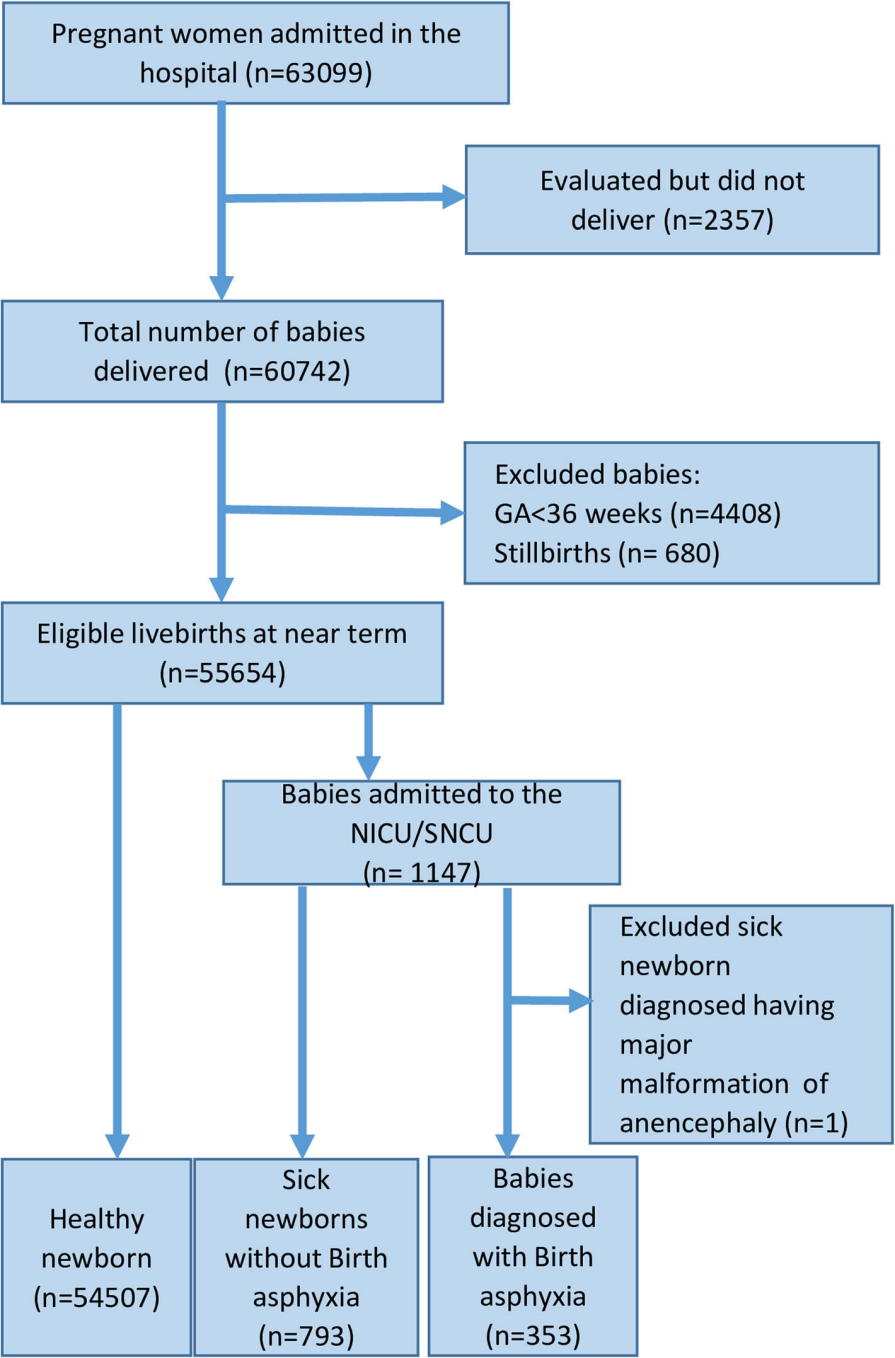
Strobe flow diagram

During the study period, there were 63,099 pregnant women admitted and a total of 60,742 deliveries were conducted in the hospitals. A total of 54,492 livebirths, who were born at term (≥37 weeks of gestation) were recruited in the study. A total of 341 babies were diagnosed as BA (Fig. 1).

The incidence of BA was 6 per 1000 near term live births. The incidence was higher (8 per 1000) among women of 35 years or above. Babies born to Muslim community had higher incidence of BA (8 per 1000) than other ethnic groups (Table 2). The obstetric factors such as induction of labour, mode of delivery, suspected maternal infection, fetal distress, prolonged labour, malposition and prolapsed cord were all found to be associated with BA on univariate analysis (Table 3).

**Table 2:** Incidence of Birth Asphyxia (*n* = 54,492)

	Incidence of Birth Asphyxia N (per 1000 livebirths)	Total term livebirths N (%)
Overall	341 (6)	54,492 (100)
By maternal age		
15–19 years	52 (7)	7288 (13.4)
20–34 years	278 (6)	45,843 (84.1)
35 years and above	11 (8)	1361 (2.5)
By ethnicity		
Dalit	58 (6)	9329 (17.2)
Janajati	91 (6)	15,774 (29.1)
Madhesi	20 (5)	3913 (7.2)
Muslim	11 (8)	1367 (2.5)
Chhetri/Brahmin	148 (7)	21,592 (39.9)
Others	13 (6)	2176 (4.0)

**Table 3:** Obstetric and neonatal factors associated with Birth Asphyxia (*n =* 54,492)

Variables	BA (***n =*** 341) N (%)	others (***n =*** 54,151) N (%)	cOR (95% CI)	aOR (95% C.I.) ^**a**^
**Induction of labour**				
No induction (spontaneous)	260 (76.2)	37,852 (69.9)	Reference	Reference
Induction with prostaglandins	48 (14.1)	9574 (17.7)	0.7 (0.5–0.9)	0.7 (0.5–1.0)
Induction with artificial rupture of membrane	14 (4.1)	1387 (2.6)	1.5 (0.9–2.5)	1.5 (0.9–2.6)
Induction with oxytocin	19 (5.6)	5338 (9.9)	0.5 (0.3–0.8)	0.5 (0.3–0.9)
**Mode of Delivery**				
Spontaneous Vaginal Delivery	201 (58.9)	40,656 (75.1)	Reference	Reference
Instrumental Delivery	45 (13.2)	1902 (3.5)	4.8 (3.5–6.6)	4.4 (3.1–6.1)
Caesarian Section	95 (27.9)	11,593 (21.4)	1.7 (1.3–2.1)	1.2 (0.9–1.6)
**Complication during labour and delivery**				
Multiple Delivery	2 (0.6)	201 (0.4)	1.6 (0.4–6.4)	1.2 (0.3–4.8)
Suspected maternal infection	80 (23.5)	9129 (16.9)	1.5 (1.2–1.9)	1.4 (0.9–1.9)
Fetal distress in labour	10 (2.9)	771 (1.4)	2.1 (1.1–3.9)	1.9 (1.0–3.6)
Prolonged labour	7 (2.1)	493 (0.9)	2.3 (1.1–4.8)	2.0 (0.9–4.3)
Malpresentation /malposition	18 (5.3)	1440 (2.7)	2.0 (1.3–3.3)	1.8 (1.0–3.0)
Prolapsed cord	1 (0.3)	17 (0.0)	9.4 (1.2–70.6)	3.4 (0.4–30.8)
Meconium aspiration	18 (5.3)	91 (0.2)	33.1 (19.7–55.5)	23.7 (13.8–40.9)
**Birth weight (grams)**				
2500–4000	301 (88.3)	50,398 (93.1)	Reference	Reference
< 2500	27 (7.9)	2452 (4.5)	1.8 (1.2–2.7)	2.0 (1.3–2.9)
> 4000	13 (3.8)	1301 (2.4)	1.7 (1.0–2.9)	1.3 (0.8–2.4)
**Gestational age (weeks)**				
37–41 weeks	321 (94.1)	52,712 (97.3)	Reference	Reference
≥ 42 weeks	20 (5.9)	1439 (2.7)	2.3 (1.5–3.6)	2.0 (1.3–3.3)
**Sex of the baby**				
Female	118 (34.6)	24,735 (45.7)	Reference	Reference
Male	223 (65.4)	29,416 (54.3)	1.6 (1.3–2.0)	1.6 (1.2–2.0)

^a^adjusted for all the variables

### Maternal and perinatal risk factors

In multivariate analysis, babies born with instrumented delivery had more than four times the risk of developing BA than those with spontaneous vaginal delivery (aOR:4.4, 95% CI, 3.1–6.1). Fetal distress during labour resulted in an 90% increased risk of BA (aOR:1.9, 95% CI, 1.0–3.6) and malposition resulted in 80% increased risk (aOR:1.8, 95% CI, 1.0–3.0). Babies weighing < 2500 g had a 2-fold increased risk of having BA (aOR:2.0, 95% CI, 1.3–2.9) compared to babies with normal birth weight of 2500–4000 g. Babies with a gestational age of ≥42 weeks had 2-fold increased risk of having BA (aOR:2.0, 95% CI, 1.3–2.9) when compared to babies of term gestational age. Male babies had a 60% increased risk of developing BA (aOR:1.6, 95% CI, 1.2–2.0). Babies who had meconium aspiration had a 24-fold risk of developing BA (aOR:23.7, 95% CI, 13.8–40.9) (Table 3).

### Outcomes

Babies with BA had 43 times higher risk of pre-discharge mortality (aOR:42.6, 95% CI, 32.2–56.3) as compared to babies who did not have BA (Table 4).

**Table 4:** Pre-discharge mortality associated with babies with Birth Asphyxia (*n* = 54,492)

Variables	Pre-discharge mortality (***n*** = 458) N (%)	Alive at discharge (***n*** = 54,034) N (%)	cOR (95% CI)	aOR (95% C.I.) ^**a**^
**Others**	377 (82.3%)	53,774 (99.5%)	Reference	Reference
**BA**	81 (17.7%)	260 (0.5%)	44.4 (33.9–58.2)	42.6 (32.2–56.3)

^a^adjusted for gestational age, birth weight and sex of the baby

## Discussion

This study describes the incidence of BA in term livebirths in Nepal based on the information from 12 selected hospitals across the country. In this study, the incidence of BA was estimated to be 6 per 1000 term livebirths. Neonates born with instrumental delivery had a greater risk of developing BA as compared to spontaneous vaginal delivery in this study. Neonates who were born to mothers who had fetal distress during labour and mal-presentation had risk for BA. Neonates who had birth weight less than 2500 g had twice fold risk for BA than those who had 2500–4000 g. Neonates born at gestational age 42 weeks or more had twice fold risk of BA than those who had gestational age 37–41 weeks. Male neonates had higher risk to BA than female neonates.

The overall incidence of BA reported in developed countries ranges from 1.6 to 24% varying by the type of study (single or multicenter or population based) and the operational definition of BA [22–24]. Multi-centric study in South India reported incidence of 24% [25] and 36% in a single center study in Nepal [26]. Similar, incidence of BA has been reported from systematic reviews and meta-analysis in Africa range from 15.9 to 22.3% [12, 23, 27]. The wide range in reported incidence could be due to variation in quality of intrapartum and perinatal care.

A single centered study in Ethopia reported prolonged labour, cesarean section delivery, meconium stained amniotic fluid (AF), fetal distress, and low birth weight as factors associated with BA [22]. A study conducted in Thailand reported a significantly higher risk for BA with vacuum extraction [28]. Babies with a gestational age of ≥42 weeks had higher of having BA similar to our results [28]. Instrumented delivery poses a risk for prolonged hypoxia, trauma and intracranial bleeds, all of which may contribute to CNS injury and clinical features of BA [22]. The rate of instrumentation is lower in public hospitals than in higher settings and represent complicated deliveries, explaining higher rate of association noted with this factor [23].

Both low birth weight and post term babies may have higher chances of perinatal complications due to placental insufficiency with the added risk factors of larger size in post-dated infants [29]. While gestational dating is based on last menstrual period (LMP) in our dataset, these known risk factors for BA were identified in our study as well. In evaluating neonatal data, we identified a higher risk of BA in male babies. Gender difference in microglial activation, inflammation and immature immune response have been suggested to influence outcomes with BA, however, we would not anticipate gender influencing maternal or obstetric factors and the actual risk of BA [30].

Finally, babies with meconium aspiration were at higher risk of developing BA in this study. A case-control study of hypoxic-ischemic encephalopathy in newborn infants at > 36 weeks gestation found that higher grade meconium was significantly associated with BA [31]. Intrauterine meconium release can complicate respiratory status at birth with risk of profound hypoxia resulting in BA [32]. Meconium aspiration in our dataset conferred the highest of all risks, with over a 20-fold increased adjusted risk.

Notably, our study identified, that pre-discharge mortality rates associated with BA was quite high at 17%. However, this mortality rate is lower than observed in a previous study on outcomes of BA in Nepal in which a 31% mortality was identified [33]. A study in Nigeria showed that the mortality rate was more than 30% among neonates who had birth asphyxia [13]. A better treatment modality based on the severity of BA may reduce the risk of mortality among infant impacted. As example, therapeutic hypothermia reduces long-term mortality in moderate to severe BA by about 25% [9] and is not currently in use in Nepal.

### Limitations and strengths

This study has numerous limitations. First, this study doesn’t report BA rates for preterm infants and only includes near-term babies. Second, limited data is available on outcomes, most notably post discharge data on rates of morbidity and mortality. Finally, not all women consented to be part of the study and the out-born babies were not included, which might bias the incidence rate.

Nevertheless, this study has several strengths. This study has a multi-centric design covering a large number of deliveries. Newborns suffering from complications are transferred to SNCU/NICU and it is likely that all infants with moderate or severe BA would be captured in our data set from there.

## Conclusion

This study provides incidence of Birth Asphyxia (BA) babies born in public referral hospitals of Nepal as well as the associated obstetric and neonatal risk factors and mortality outcomes. Improving management of high-risk women during labour and delivery can reduce the risk of perinatal events and development of BA. Further, advancing the management of infants with BA in the public hospital through specialised care may reduce the risk of associated mortality due to BA.

## Data Availability

The datasets used/or analysed during the current study are available from the corresponding author on reasonable request.

## References

[1] Lee AC, Cousens S, Wall SN, Niermeyer S, Darmstadt GL, Carlo WA, Keenan WJ, Bhutta ZA, Gill C, Lawn JE (2011). Neonatal resuscitation and immediate newborn assessment and stimulation for the prevention of neonatal deaths: a systematic review, meta-analysis and Delphi estimation of mortality effect. BMC Public Health.

[2] Wall SN, Lee AC, Niermeyer S, English M, Keenan WJ, Carlo W, Bhutta ZA, Bang A, Narayanan I, Ariawan I, Lawn JE. Neonatal resuscitation in low-resource settings: what, who, and how to overcome challenges to scale up? Int J Gynaecol Obstet. 2009;107(Suppl 1):S47-62, S63-4. 10.1016/j.ijgo.2009.07.013 PMC2875104 19815203

[3] Kc A, Lawn JE, Zhou H, Ewald U, Gurung R, Gurung A, et al. Not Crying After Birth as a Predictor of Not Breathing. Pediatrics. 2020;145(6). 10.1542/peds.2019-2719 32398327

[4] Hooper SB, Polglase GR, te Pas AB (2015). A physiological approach to the timing of umbilical cord clamping at birth. Arch Dis Child Fetal Neonatal Ed.

[5] Lee AC, Kozuki N, Blencowe H, Vos T, Bahalim A, Darmstadt GL, Niermeyer S, Ellis M, Robertson NJ, Cousens S (2013). Intrapartum-related neonatal encephalopathy incidence and impairment at regional and global levels for 2010 with trends from 1990. Pediatr Res.

[6] Liu L, Oza S, Hogan D, Chu Y, Perin J, Zhu J, Lawn JE, Cousens S, Mathers C, Black RE (2016). Global, regional, and national causes of under-5 mortality in 2000-15: an updated systematic analysis with implications for the sustainable development goals. Lancet.

[7] Kurinczuk JJ, White-Koning M, Badawi N (2010). Epidemiology of neonatal encephalopathy and hypoxic-ischaemic encephalopathy. Early Hum Dev.

[8] Herrera-Marschitz M, Neira-Pena T, Rojas-Mancilla E, Morales P, Bustamante D, Leyton L, Gebicke-Haerter P (2015). Short- and long-term consequences of perinatal asphyxia: looking for neuroprotective strategies. Adv Neurobiol.

[9] Jacobs SE, Berg M, Hunt R, Tarnow-Mordi WO, Inder TE, Davis PG (2013). Cooling for newborns with hypoxic ischaemic encephalopathy. Cochrane Database Syst Rev.

[10] Lawn JE, Lee AC, Kinney M, Sibley L, Carlo WA, Paul VK, Pattinson R, Darmstadt GL (2009). Two million intrapartum-related stillbirths and neonatal deaths: where, why, and what can be done?. Int J Gynaecol Obstet.

[11] Igboanugo S, Chen A, Mielke JG (2020). Maternal risk factors for birth asphyxia in low-resource communities. A systematic review of the literature. J Obstet Gynaecol.

[12] Sendeku FW, Azeze GG, Fenta SL (2020). Perinatal asphyxia and its associated factors in Ethiopia: a systematic review and meta-analysis. BMC Pediatr.

[13] Egharevba OI, Kayode-Adedeji BO, Alikah SO (2018). Perinatal asphyxia in a rural Nigerian hospital: incidence and determinants of early outcome. J Neonatal Perinatal Med.

[14] Bhutta ZA, Das JK, Bahl R, Lawn JE, Salam RA, Paul VK, Sankar MJ, Blencowe H, Rizvi A, Chou VB (2014). Can available interventions end preventable deaths in mothers, newborn babies, and stillbirths, and at what cost?. Lancet.

[15] Lindstrom K, Hallberg B, Blennow M, Wolff K, Fernell E, Westgren M (2008). Moderate neonatal encephalopathy: pre- and perinatal risk factors and long-term outcome. Acta Obstet Gynecol Scand.

[16] Goldenberg RL, McClure EM (2009). Reducing intrapartum stillbirths and intrapartum-related neonatal deaths. Int J Gynaecol Obstet.

[17] Lee AC, Cousens S, Darmstadt GL, Blencowe H, Pattinson R, Moran NF, Hofmeyr GJ, Haws RA, Bhutta SZ, Lawn JE (2011). Care during labor and birth for the prevention of intrapartum-related neonatal deaths: a systematic review and Delphi estimation of mortality effect. BMC Public Health.

[18] Kc A, Ewald U, Basnet O, Gurung A, Pyakuryal SN, Jha BK, Bergstrom A, Eriksson L, Paudel P, Karki S (2019). Effect of a scaled-up neonatal resuscitation quality improvement package on intrapartum-related mortality in Nepal: a stepped-wedge cluster randomized controlled trial. PLoS Med.

[19] Chaulagain DR, Malqvist M, Brunell O, Wrammert J, Basnet O, Kc A (2021). Performance of health workers on neonatal resuscitation care following scaled-up quality improvement interventions in public hospitals of Nepal - a prospective observational study. BMC Health Serv Res.

[20] Lindback C, Kc A, Wrammert J, Vitrakoti R, Ewald U, Malqvist M (2014). Poor adherence to neonatal resuscitation guidelines exposed; an observational study using camera surveillance at a tertiary hospital in Nepal. BMC Pediatr.

[21] Kc A, Bergstrom A, Chaulagain D, Brunell O, Ewald U, Gurung A, Eriksson L, Litorp H, Wrammert J, Gronqvist E (2017). Scaling up quality improvement intervention for perinatal care in Nepal (NePeriQIP); study protocol of a cluster randomised trial. BMJ Glob Health.

[22] Wosenu L, Worku AG, Teshome DF, Gelagay AA (2018). Determinants of birth asphyxia among live birth newborns in University of Gondar referral hospital, Northwest Ethiopia: a case-control study. PLoS One.

[23] Workineh Y, Semachew A, Ayalew E, Animaw W, Tirfie M, Birhanu M (2020). Prevalence of perinatal asphyxia in east and Central Africa: systematic review and meta-analysis. Heliyon.

[24] Wood S, Crawford S, Hicks M, Mohammad K (2021). Hospital-related, maternal, and fetal risk factors for neonatal asphyxia and moderate or severe hypoxic-ischemic encephalopathy: a retrospective cohort study. J Matern Fetal Neonatal Med.

[25] Ebenezer ED, Londhe V, Rathore S, Benjamin S, Ross B, Jeyaseelan L, Mathews JE (2019). Peripartum interventions resulting in reduced perinatal mortality rates, and birth asphyxia rates, over 18 years in a tertiary Centre in South India: a retrospective study. BJOG.

[26] Manandhar SR, Basnet R (2019). Prevalence of perinatal asphyxia in neonates at a tertiary care hospital: a descriptive cross-sectional study. JNMA J Nepal Med Assoc.

[27] Bruckmann EK, Velaphi S (2015). Intrapartum asphyxia and hypoxic ischaemic encephalopathy in a public hospital: incidence and predictors of poor outcome. S Afr Med J.

[28] Futrakul S, Praisuwanna P, Thaitumyanon P (2006). Risk factors for hypoxic-ischemic encephalopathy in asphyxiated newborn infants. J Med Assoc Thail.

[29] Kumar S, Paterson-Brown S (2010). Obstetric aspects of hypoxic ischemic encephalopathy. Early Hum Dev.

[30] Mirza MA, Ritzel R, Xu Y, McCullough LD, Liu F (2015). Sexually dimorphic outcomes and inflammatory responses in hypoxic-ischemic encephalopathy. J Neuroinflammation.

[31] Hayes BC, McGarvey C, Mulvany S, Kennedy J, Geary MP, Matthews TG, King MD (2013). A case-control study of hypoxic-ischemic encephalopathy in newborn infants at >36 weeks gestation. Am J Obstet Gynecol.

[32] Milsom I, Ladfors L, Thiringer K, Niklasson A, Odeback A, Thornberg E (2002). Influence of maternal, obstetric and fetal risk factors on the prevalence of birth asphyxia at term in a Swedish urban population. Acta Obstet Gynecol Scand.

[33] Lee AC, Mullany LC, Tielsch JM, Katz J, Khatry SK, LeClerq SC, Adhikari RK, Darmstadt GL (2011). Incidence of and risk factors for neonatal respiratory depression and encephalopathy in rural Sarlahi, Nepal. Pediatrics.

